# An introduction to Iranian Collembola (Hexapoda): an update to the species list

**DOI:** 10.3897/zookeys.335.5491

**Published:** 2013-09-25

**Authors:** Masoumeh Shayanmehr, Elliyeh Yahyapour, Morteza Kahrarian, Elham Yoosefi Lafooraki

**Affiliations:** 1 Department of Plant Protection, Faculty of Crop Sciences, Sari University of Agricultural Sciences and Natural Resources, Sari, Mazandaran, Iran, Po. Box. 578; 2 Kermanshah Branch, Islamic Azad University, Kermanshah, Iran. Imam Khomeini Campus, Islamic Azad University, Farhikhtegan Bld., ShahidJ’afari St., Kermanshah, Iran

**Keywords:** Springtails, taxonomy, Iran, checklist

## Abstract

The Collembola fauna of Iran is little known and no comprehensive examination of this group of Hexapoda is available for this region. The only notable work on Collembola was carried out by Cox (1982). Recently, studies on the Collembola fauna have started in several regions. In this paper, publications by different researchers are documented and the species that have been found in different regions of Iran until January 2013 are listed. At present, 112 species, belonging to 18 families and 57 genera are known from Iran.

## Introduction

Worldwide about 8,000 species of Collembola have been described (Bellinger et al. 1996–2012). Most Collembola species have been reported from Europe or North America but little literature or taxonomic keys are available from Asian countries especially from those of the Middle East.

Iran is a country in southwest Asia with an area of 1,648,195 km^2^. It spans several different climatic zones and biomes, therefore the diversity of animals is high and this presumably also applies to those living in soil. According to biogeographic zonation, Iran has been considered part of the Palearctic region by most authors. However, some parts in the southern Iran, such as Khuzestan Plain and Persian Gulf coast, have been considered as Ethiopian (Cox 1982). The study of Collembola fauna of Iran is poor and most attention has been paid to crop pest species, which are few.

Farrahbakhsh (1961) was the first to provide information on Iranian Collembola. He reported *Sminthurus viridis* Linnaeus, 1758 from wheat and alfalfa fields in Khuzestan (Southern Iran). The most comprehensive study on Collembola was carried out by Cox (1982) who travelled to Northern, West and Central provinces in Iran and collected and identified 70 species of 30 genera and five families. The scientific names of some species recorded by Cox were changed later and their modern names are used in Appendix: *Cyphoderus ambigua* was changed to *Oncopodura ambigua* according to Christiansen (1957); the genus *Cryptopygus* Willem, 1902 was changed to *Hemisotoma* Börner, 1903 according to Rusek (2000). The species *Folsomia litsteri* Bagnall, 1939 was changed to *Folsomia candida* (Willem, 1902); *Folsomia multiseta* Stach, 1947 was changed to *Folsomia penicula* Bagnall, 1939; *Neanura echinata* (Kos, 1940) was changed to *Thaumanura echinata* (Kos, 1940); the genus *Xenyllodes* Axelson, 1903 was changed to *Axenyllodes* Stach, 1949; *Xenyllodes lamellifera* was changed to *Superodontella lamellifera* (Axelson, 1903); *Onychiurus pseudogranulosus* Gisin, 1951 was changed to *Onychiuroides pseudogranulosus* (Gisin, 1951); *Onychiurus rectopapillatus* Stach, 1933 was changed to *Orthonychiurus rectopapillatus* (Stach, 1933). Also *Sminthurus marginatus* Schött, 1893 which is recorded by Kahrarian et al. (2012) was changed to *Caprainea marginata* (Schoett, 1893). Recently, some master and doctoral students started to work on the Collembola fauna of several regions of Iran and therefore the list of species certainly will increase.

Here we provide an update to the list of Iranian Collembola published from 1961 to 2013 mainly from the northern Iran. Obviously, the fauna of large parts of Iran is unknown and there is a need for additional research on the distribution of species but also on other aspects of these animals such as ecology, biology and their role in ecological processes in different ecosystems. It is the intention of the paper to encourage young entomologists to become aware of these gaps of knowledge and direct their interest towards Collembola fauna of this country.

## Methods

**Figure 1.**
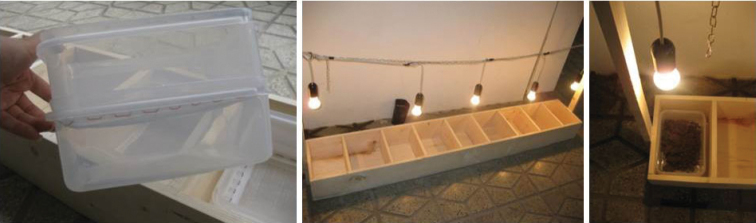
Extractor system for soil animals.

The updated Iranian Collembola list was provided from two resources. First, it is based on bibliographic references and unpublished records from different regions (taxa not ascribed to species are not included in the total number of species). Second, results of sampling campaigns of Collembola by authors from different regions in Iran during 2009–2012 years are included. In the latter studies, soil and litter samples were collected from various habitats in Sari, Gorgan (Northern Iran) and Kermanshah (Western Iran). The samples were placed in dark polythene bags. Collembola were extracted from soil and leaf litter by Berlese funnels (Figure 1). Animals were collected in water and separated under a dissecting microscope. The extracted specimens were preserved in 75% ethanol. Permanent microscopic slides were prepared using Hoyer medium; for immediate identification, a mixture of lactic acid and glycerin (5:1) was used. For observing detailed structures of specimens, a 100×oil immersion objective was used. The specimens were identified by taxonomic keys such as Gisin (1960), Fjellberg (1980, 1998, and 2007), Bretfeld (1999) and Potapov (2001). Identification of species was confirmed by Collembola experts such as Dr. Mikhail Potapov (Russia), Dr. Hans-Uergen Schulz (Germany), Dr. Ulrich Burkhart (Germany) and Dr. Louis Deharveng (France).

## Result and discussion

**Figure 2.**
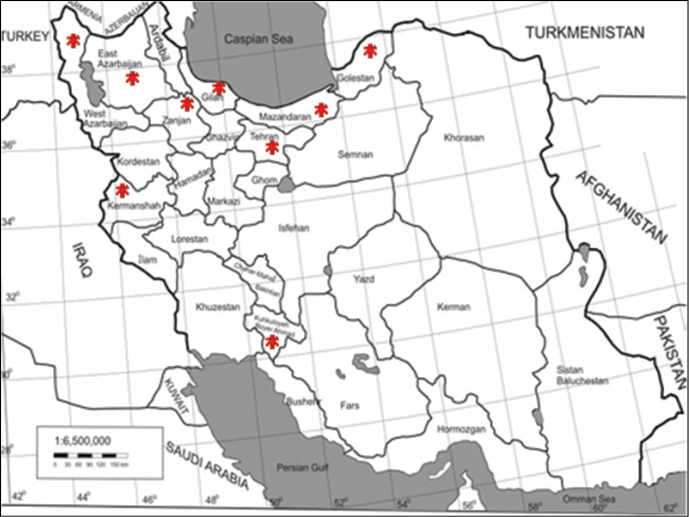
Map of Iran showing the provinces (*) from which Collembola have been collected.

**Figure 3.**
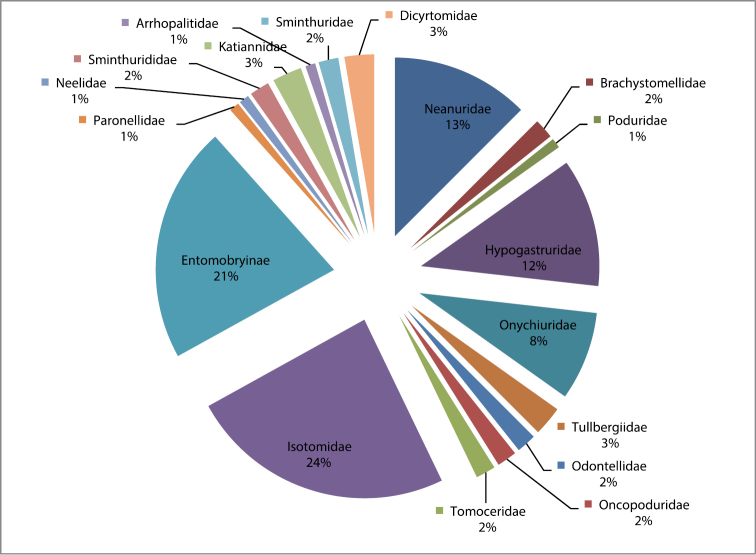
Percentage of Iranian Collembola species from different families.

The number of Iranian Collembola species recorded until March 2012 is 112, belonging to 18 families and 57 genera. A systematic list of species according to the modern classification for the class Collembola (Deharveng, 2004) and details of species collection are shown in Appendix. The distribution of the species in the different provinces is shown in Figure 2. Most recorded species belong to Isotomidae (24%) and Entomobryidae (21%) (Figure 3). Collembola taxa for which species were not identified and which were reported as sp. was not included in this checklist. In addition to the genera shown in Appendix, some specimens from the genera, *Pachyotoma* (Bagnall, 1949), *Prodrepanura* (Stach, 1963), *Isotomodes* (Linnaniemi, 1907), *Gnathofolsomia* (Deharveng & Christian, 1984), *Protaphorura* (Absolon, 1901) (distributed in Kermanshah) by Kahrarian et al. (2012), *Orchesella* Templeton, 1835 (distributed in Kermanshah and Golestan) by Falahati et al. (2011), *Stenacidia* Reuter, 1881, (distributed in Gilan) by Daghighi (2012) were reported for Iran fauna but the species were not identified. Some species belonging to Symphypleona were recorded by Falahati et al. (2013b) with dubious identification, for example the photo illustrated the species *Sminthurinus reticulatus* seems to be belonging to Arrhopalitdae. The species listed in this paper include *Dicyrtomina ornate*, *Sminthurides aquaticus*, *Smynthurinus signatus*, *Smynthurinus transvernalis*, *Smynthurinus reticulatus* and *Smynthurinus elegans*. Additionally Dr. Bretfeld didn’t confirm the identifications. For these reasons the species recorded in this paper are excluded from present checklist.

The results of this paper indicate that study of Collembola is at an early stage in Iran but recently interest in the group is increasing.

## Appendix

**Table:** Checklist of Iranian Collembola species. Species marked by * were recorded for the first time from Iran.

**Taxonomy: Class/Order/Family/Subfamily/Species**	**Reference**	**Distribution in Iran, Province/Location**	**Habitat**
**Class Collembola**			
**Order Poduromorpha**			
**Family Neanuridae**			
**Subfamily Pseudachorutinae**			
**Genus *Anurida* Laboulbne, 1865**			
*Anurida ellipsoides* Stach, 1949	Cox (1982)	E. Azarbaijan, W. Azarbaijan	Soil, Leaf litter
*Anurida sensillata* Gisin, 1953	Cox (1982)	Gilan	Soil, Leaf litter
*Anurida thalassophila* (Bagnall, 1939)	Cox (1982)	Gilan, E. Azarbaijan	Soil, Leaf litter
**Genus *Pseudachorutes* Tullberg, 1871**			
*Pseudachorutes dubius* Krausbauer, 1898	Cox (1982)	Central, Gilan	Soil, Leaf litter
*Pseudachorutes parvulus* Börner, 1901	Cox (1982)	Central, Gilan	Soil, Leaf litter
*Pseudachorutes subcrassus* Tullberg, 1871	Cox (1982)	Central, Gilan	Soil, Leaf litter
**Subfamily Frieseinae**			
**Genus *Friesea* Dalla Torre, 1895**			
*Friesea mirabilis* (Tullberg, 1871)	Cox (1982)	Central, Gilan, E. Azarbaijan	Soil, Leaf litter
**Subfamily Morulininae**			
**Genus *Morulina* Börner, 1906**			
*Morulina verrucosa* Börner, 1903*	Daghighi (2012)	Gilan/Rasht	Soil (*Ulmus* sp.)
**Subfamily Neanurinae**			
**Genus *Bilobella* Caroli, 1912**			
*Bilobella aurantiaca* (Caroli, 1912)	Cox (1982)	Central, Mazandaran, Gilan, E. Azarbaijan	Soil, Leaf litter
**Genus *Cryptonura* Cassagnau, 1979**			
*Cryptonura persica* sp. n.*	Smolis et al. (2012)		
*Cryptonura maxima* sp. n.*	Smolis et al. (2012)		
**Genus *Deutonura* Cassagnau, 1979**			
*Deutonura decolorata* (Gama & Gisin, 1964)	Cox (1982)	Mazandaran	Soil, Leaf litter
**Genus *Neanura* MacGillivray, 1893**			
*Thaumanura echinata* (Kos, 1940)	Cox (1982)	unknown	Soil, Leaf litter
*Neanura muscorum* (Templeton, 1835)	Cox (1982), Yahyapour (2012)	Mazandaran, Gilan, Zanjan, Mazandaran/Sari	Leaf litter
**Family Brachystomellidae**			
**Genus *Brachystomella* Agren, 1903**			
*Brachystomella parvula* (Schäffer, 1896)	Cox (1982)	Gilan, E. Azarbaijan, Zanjan	Soil, Leaf litter
*Brachystomella nubila* Gisin, 1957	Cox (1982)	Gilan, E. Azarbaijan, Zanjan	Soil, Leaf litter
**Family Poduridae**			
**Genus *Podura* Linnaeus, 1758**			
*Podura aquatica* Linnaeus, 1758	Cox (1982)	Central	Soil, Leaf litter near water
**Family Hypogastruridae**			
**Genus *Ceratophysella* Börner, 1932**			
*Ceratophysella armata* (Nicolet, 1841)*	Falahati et al. (2012)	Kohgiluyeh and Boyer-Ahmad, Charam	Soil
*Ceratophysella denticulata* (Bagnall, 1941)	Cox (1982), Yahyapour (2012), Ghahramaninezhad et al. (2012)	Central, Mazandaran, Gilan, E. Azarbaijan, W. Azarbaijan, Zanjan Mazandaran/Sari, Kermanshah	Leaf litter, Soil
*Ceratophysella stercoraria* Stach, 1963*	Kahrarian et al. (2012), Falahati et al. (2012)	Kermanshah, Sahneh, Kohgiluyeh and Boyer-Ahmad, Charam	Soil
**Genus *Choreutinula* Paclt, 1944**			
*Choreutinula inermis* Tullberg, 1871*	Daghighi (2012)	Gilan/Rasht	Soil (*Prunus* sp., *Ficus* sp., *Platanus* sp., *Ulmus* sp., *Alnus* sp.)
**Genus *Hypogastrura* Bourlet, 1839**			
*Hypogastrura assimilis* (Krausbauer, 1898)*	Falahati et al. (2012)	Kohgiluyeh and Boyer-Ahmad, Charam	Soil
*Hypogastrura manubrialis* (Tullberg, 1869)	Cox (1982), Falahati et al. (2012)	Central, Mazandaran, Gilan, E. Azarbaijan, W. Azarbaijan, Zanjan, Kohgiluyeh and Boyer-Ahmad, Charam	Soil, Leaf litter
*Hypogastrura tullbergi* (Schäffer, 1900)	Cox (1982)	Mazandaran, Gilan	Soil, Leaf litter
*Hypogastrura vernalis* (Carl, 1901)*	Falahati et al. (2012)	Kohgiluyeh and Boyer-Ahmad, Charam	Soil
**Genus *Xenylla* Tullberg, 1869**			
*Xenylla humicola* (Fabricius, 1780)	Cox (1982)	Gilan, E. Azarbaijan, W. Azarbaijan	Soil, Leaf litter
*Xenylla maritima* Tullberg, 1869	Cox (1982)	Gilan, E. Azarbaijan, W. Azarbaijan	Soil, Leaf litter
*Xenylla welchi* Folsom, 1916*	Yahyapour (2012), Falahati et al. (2012)	Mazandaran/Sari, Kohgiluyeh and Boyer-Ahmad, Charam	Leaf litter, Soil
**Genus *Willemia* Börner, 1901**			
*Willemia anophthalma* Börner, 1901	Cox (1982)	Central, E. Azarbaijan	Soil, Leaf litter
*Willemia aspinata* Stach, 1949*	Daghighi (2012)	Gilan/Rasht	Soil (*Pinus* sp.)
**Family Onychiuridae**			
**Subfamily Onychiurinae**			
**Genus *Protaphorura* Absolon, 1901**			
*Protaphorura fimata* (Gisin, 1952)	Cox (1982)	Central, Mazandaran, Gilan, E. Azarbaijan, W. Azarbaijan, Zanjan	Soil, Leaf litter
*Protaphorura bicampata* (Gisin, 1956)	Cox (1982)	Central, Mazandaran, Gilan, E. Azarbaijan, W. Azarbaijan, Zanjan	Soil, Leaf litter
*Protaphorura quadriocellata* (Gisin, 1947)	Cox (1982)	Central, Mazandaran, Gilan, E. Azarbaijan, W. Azarbaijan, Zanjan	Soil, Leaf litter
**Genus *Onychiuroides* Bagnall, 1948**			
*Onychiuroides granulosus* (Stach, 1930)	Cox (1982)	Central, Mazandaran, Gilan, E. Azarbaijan, W. Azarbaijan, Zanjan	Soil, Leaf litter
*Onychiuroides pseudogranulosus* (Gisin, 1951)	Cox (1982)	Central, Mazandaran, Gilan, E. Azarbaijan, W. Azarbaijan, Zanjan	Soil, Leaf litter
**Genus *Hymenaphorura* Gervais, 1841**			
*Hymenaphorura sibirica* (Tullberg, 1876)	Cox (1982)	Central, Mazandaran, Gilan, E. Azarbaijan, W. Azarbaijan, Zanjan	Soil, Leaf litter
**Genus *Orthonychiurus* Stach, 1954**			
*Orthonychiurus folsomi* (Schäffer, 1900)*	Yahyapour (2012)	Mazandaran/Sari	Leaf litter
*Orthonychiurus rectopapillatus* (Stach, 1933)	Cox (1982)	Central, Mazandaran, Gilan, E. Azarbaijan, W. Azarbaijan, Zanjan	Soil, Leaf litter
**Subfamily Tetrodontophorinae**			
**Genus *Tetrodontophora* Reuter, 1882**			
*Tetrodontophora bielanensis* (Waga, 1842)*	Daghighi (2012)	Gilan/Rasht	Soil (*Ulmus* sp., *Prunus* sp., *Alnus* sp., *Parrotia persica*, *Acer* sp., *Salix alba*, *Punica * sp., *Robinia* sp., *Eriobotrya* sp., *Populus* sp.)
**Family Tullbergiidae**			
**Genus *Mesaphorura* Börner, 1901**			
*Mesaphorura krausbaueri* (Börner, 1901)	Cox (1982)	Central, Mazandaran, Gilan, E. Azarbaijan, Zanjan	Soil, Leaf litter
**Genus *Metaphorura* Stach, 1954**			
*Metaphorura affinis* Börner, 1902	Cox (1982), Daghighi (2012), Ghahramaninezhad et al. (2012)	Central, Gilan, E. Azarbaijan, Gilan/Rasht, Kermanshah	Soil (*Quercus* sp., *Platanus* sp., *Alnus* sp, *Punica* sp., *Ulmus* sp., *Pinus* sp., *Cupressus* sp., *Robinia* sp., *Eriobotrya* sp., *Zelkova* sp.) litter
**Genus *Paratullbergia* Womersley, 1930**			
*Paratullbergia callipygos* (Börner, 1902)	Cox (1982)	Central, Zanjan	Soil, Leaf litter
**Family Odontellidae**			
**Genus *Axenyllodes* Stach, 1949**			
*Axenyllodes bayeri* Kseneman, 1935	Cox (1982)	Gilan, E. Azarbaijan	Soil, Leaf litter
**Genus *Superodontella* Stach, 1949**			
*Superodontella lamellifera* (Axelson, 1903)	Cox (1982)	E. Azarbaijan	Soil, Leaf litter
**Order Entomobryomorpha**			
**Family Oncopoduridae**			
**Genus *Oncopodura* Carl & Lebedinsky, 1905**			
*Oncopodura ambigua* Christiansen, 1957	Cox (1982)	Mazandaran, Gilan	Soil, leaf litter
*Oncopodura hamata* Carl & Lebedinsky, 1905*	Daghighi (2012)	Gilan/Rasht	Soil (*Ulmus* sp., *Prunus* sp.)
**Family Tomoceridae**			
**Genus *Tomocerus* Nocolet, 1842**			
*Tomocerus minor* (Lubbock, 1862)	Cox (1982)	Central, Mazandaran, Gilan, E. Azarbaijan, Zanjan	Soil, Leaf litter
*Tomocerus vulgaris* (Tullberg, 1871)	Cox (1982)	Central, Mazandaran, Gilan, E. Azarbaijan, Zanjan	Soil, Leaf litter
**Family Isotomidae**			
**Genus *Anurophorus* Nocolet, 1842**			
*Anurophorus coiffaiti* Cassagnau & Delamare, 1955*	Falahati (2012), Falahati et al. (2013a), Daghighi (2012), Daghighi et al. (2013a), Daghighi et al. (2013b)	Golestan/Gorgan, Gilan/Rasht	Soil (*Ulmus* sp., *Punica* sp.)
**Genus *Ballistura* Börner, 1906**			
*Ballistura schoetti* (Dalla Torre, 1895)	Cox (1982)	Gilan	Soil, Leaf litter
**Genus *Hemisotoma* Bagnall, 1949**			
*Hemisotoma thermophila* (Axelson, 1900)	Cox (1982), Daghighi (2012), Daghighi et al. (2013a), Daghighi et al. (2013b)	Central, Mazandaran, Gilan, E. Azarbaijan, W. Azarbaijan, Gilan/Rasht	Soil (*Ulmus* sp., *Ficus* sp., *Ziziphus* sp., *Populus* sp.) Leaf litter
**Genus *Desoria* Nicolet, 1841**			
*Desoria tigrina* Nicolet, 1842*	Kahrarian et al. (2012)	Kermanshah	Soil
*Desoria olivacea* (Tullberg, 1871)	Cox (1982)	E. Azarbaijan	Soil, litter
**Genus *Folsomia* Willem, 1902**			
*Folsomia binoculata* (Wahlgren 1899)*	Ghahramaninezhad et al. (2012)	Kermanshah	Soil
*Folsomia brevifurca* (Bagnall, 1949)	Cox (1982)	Mazandaran	Soil, Leaf litter
*Folsomia candida* (Willem, 1902)	Cox (1982), Yahyapour (2012)	Central, Mazandaran, Gilan, E. Azarbaijan, Mazandaran/Sari	Soil, Leaf litter
*Folsomia fimetaria* (Linnaeus, 1758)	Cox (1982)	Central, Mazandaran, Gilan	Soil, Leaf litter
*Folsomia penicula* Bagnall, 1939*	Cox (1982), Falahati (2012), Falahati et al. (2013a), Daghighi (2012), Daghighi et al. (2013a), Daghighi et al. (2013b), Falahati et al. (2011)	Central, Mazandaran, E. Azarbaijan, Golestan/Gorgan, Gilan/Rasht	Soil (*Quercus* sp.)
*Folsomia quadrioculata* (Tullberg, 1871)	Cox (1982)	Central, Mazandaran, Gilan, E. Azarbaijan, W. Azarbaijan	Soil, Leaf litter
*Folsomia similis* Bagnall, 1939*	Moravvej et al. (2007), Daghighi (2012), Daghighi et al. (2013a), Daghighi et al. (2013b)	Tehran, Gilan/Rasht	Soil (*Ficus* sp., *Pinus* sp.)
**Genus *Folsomides* Stach, 1922**			
*Folsomides angularis* Axelson, 1905*	Daghighi (2012), Daghighi et al. (2013a), Daghighi et al. (2013b)	Gilan/Rasht	Soil (*Prunus* sp.)
*Folsomides marchicus* (Frenzel, 1941)*	Kahrarian et al. (2012)	Kermanshah/Sahneh/Harsin	Soil
*Folsomides parvulus* Stach, 1922	Cox (1982), Yahyapour (2012), Kahrarian et al. (2012), Daghighi (2012), Daghighi et al. (2013a), Daghighi et al. (2013b)	Central, Mazandaran, Gilan, E. Azarbaijan, W. Azarbaijan, Mazandaran/Sari, Kermanshah/Sahneh/Harsin, Gilan/Rasht	Soil (*Morus* sp.), Leaf litter
**Genus *Isotoma* Bourlet, 1839**			
*Isotoma viridis* Bourlet, 1839	Cox (1982), Yahyapour (2012)	Central, Mazandaran, E. Azarbaijan, W. Azarbaijan, Mazandaran/Sari	Soil, Leaf litter
**Genus *Isotomiella* Bagnall, 1939**			
*Isotomiella minor* (Schäffer, 1896)	Cox (1982), Moravvej et al. (2007), Yahyapour (2012), Daghighi (2012), Daghighi et al. (2013a), Daghighi et al. (2013b), Ghahramaninezhad et al. (2012)	Mazandaran, Gilan, E. Azarbaijan, Tehran, Mazandaran/Sari, Gilan/Rasht, Kermanshah	Soil (*Quercus* sp., *Prunus* sp., *Pinus* sp.) Leaf litter
**Genus Isotomina** Börner, 1903			
*Hemisotoma pontica* Stach, 1947	Cox (1982), Moravvej et al. (2007), Kahrarian et al. (2012), Yahyapour (2012)	Central, Mazandaran, Gilan, E. Azarbaijan, W. Azarbaijan, Tehran, Kermanshah, Mazandaran/Sari	Soil, Leaf litter
*Hemisotoma orientalis* Stach, 1947	Cox (1982)	Central, Mazandaran, Gilan, E. Azarbaijan, W. Azarbaijan	Soil, Leaf litter
**Genus *Isotomurus* Börner, 1903**			
*Isotomurus maculatus* (Schäffer, 1869)*	Falahati (2012), Falahati et al. (2013a), Falahati et al. (2011)	Golestan/Gorgan	Soil
*Isotomurus palustris* (Muller, 1776)	Cox (1982)	Central, Mazandaran, Gilan	Soil, Leaf litter
*Isotomurus punctiferus* Yosii, 1963*	Falahati (2012), Falahati et al. (2013a), Daghighi (2012), Daghighi et al. (2013a), Daghighi et al. (2013b)	Golestan/Gorgan, Gilan/Rasht	Soil
**Genus *Parisotoma* Bagnall, 1940**			
*Parisotoma notabilis* (Schäffer, 1896)	Cox (1982), Moravvej et al. (2007), Kahrarian et al. (2012), Daghighi (2012), Daghighi et al. (2013a), Daghighi et al. (2013b)	Central, Mazandaran, Gilan, E. Azarbaijan, W. Azarbaijan, Zanjan, Tehran, Kermanshah, Gilan/Rasht	Soil (*Platanus* sp., *Ulmus* sp., *Cupressus* sp.) Leaf litter
**Genus *Proisotoma* Börner, 1901**			
*Proisotoma minima* Absolon, 1901*	Yahyapour (2012)	Mazandaran/Sari	Leaf litter
*Proisotoma minuta* (Tullberg, 1871)	Cox (1982), Nematollahi et al. (2009)	Central, Mazandaran, Gilan, E. Azarbaijan, W. Azarbaijan	Soil, Leaf litter, African violet
*Proisotoma subminuta* Denis, 1931*	Moravvej et al. (2007), Daghighi (2012), Daghighi et al. (2013a), Daghighi et al. (2013b)	Tehran, Gilan/Rasht	Soil (*Ulmus* sp.)
*Proisotoma tenella* Reuter, 1895*	Daghighi (2012), Daghighi et al. (2013a), Daghighi et al. (2013b)	Gilan/Rasht	Soil (*Melia* sp.)
**Family Entomobryidae**			
**Subfamily Entomobryinae**			
**Genus *Entomobrya* Rondani, 1861**			
*Entomobrya atrocincta* Schött, 1986*	Yahyapour (2012), Yahyapour et al. (2011), Kahrarian et al. (2012)	Mazandaran/Sari, Kermanshah/Harsin	Leaf litter
*Entomobrya corticalis* (Nicolet, 1841)	Cox (1982)	Gilan	Soil, Leaf litter
*Entomobrya dollfusi* Denis, 1924*	Yahyapour (2012)	Mazandaran/Sari	Leaf litter
*Entomobrya lindbergi* Stach, 1960*	Daghighi (2012), Daghighi et al. (2013b), Moravvej (2003)	Gilan/Rasht, Tehran	Soil (*Alnus* sp. and *Ulmus* sp.)
*Entomobrya lanuginosa* (Nicolet, 1841)	Cox (1982)	Central, Mazandaran, Gilan, E. Azarbaijan	Soil, Leaf litter
*Entomobrya handschini* Stach, 1922*	Moravvej (2003)	Tehran	Soil
*Entomobrya multifasciata* Tullberg, 1871*	Yahyapour (2012), Yahyapour et al. (2011)	Mazandaran/ Sari	Leaf litter
*Entomobrya unostrigata* Stach, 1930*	Moravvej (2003)	Tehran	Soil
**Genus *Mesentotoma* Salmon, 1942**			
*Mesentotoma subdollfusi* Jacquemart, 1974*	Daghighi (2012), Daghighi et al. (2013b)	Gilan/Rasht	Soil (*Alnus* sp., *Ulmus* sp., *Parrotia persica*, *Acer* sp. and *Salix alba*)
**Genus *Sinella* Brook, 1882**			
*Sinella curviseta* Brook, 1882*	Moravvej (2003)	Tehran	Soil
*Sinella tenebricosa* Folsom, 1902*	Nematollahi et al. (2009)	Isfahan/Isfahan	vegetables and cucumber greenhouses
**Subfamily Lepidocyrtinae**			
**Genus *Lepidocyrtus* Bourlet, 1839**			
*Lepidocyrtus cyaneus* Tullberg, 1871	Cox (1982)	Central, Mazandaran, Gilan, E. Azarbaijan, Zanjan	Soil, Leaf litter
*Lepidocyrtus lanuginosus* (Gmelin, 1788)	Cox (1982)	Central, Mazandaran, Gilan, E. Azarbaijan	Soil, Leaf litter
*Lepidocyrtus ruber* Schött, 1902	Cox (1982)	Central, Mazandaran, Gilan, E. Azarbaijan	Soil, Leaf litter
**Genus *Pseudosinella* Schaefer, 1897**			
*Pseudosinella alba* (Packard, 1873)*	Daghighi (2012), Daghighi et al. (2013b)	Gilan/Rasht	Soil (*Quercus* sp., *Ulmus* sp., *Platanus* sp., *Alnus* sp., *Parrotia persica*, *Acer* sp. *Salix alba*, *Punica* sp.)
*Pseudosinella duodecimpunctata* Denis, 1931	Cox (1982)	E. Azarbaijan	Soil, Leaf litter
*Pseudosinella imparipunctata* Gisin, 1953	Cox (1982)	Mazandaran, Gilan, Zanjan	Soil, Leaf litter
*Pseudosinella octopunctata* Böner, 1901	Cox (1982), Yahyapour et al. (2011), Yahyapour (2012)	Central, Mazandaran, Gilan, E. Azarbaijan, W. Azarbaijan, Zanjan, Mazandaran/Sari	Soil, Leaf litter
*Pseudosinella sexoculata* (Schött, 1902)*	Ghahramaninezhad et al. (2012)	Kermanshah	Soil
**Subfamily Seirinae**			
**Genus *Seira* Lubbock, 1870**			
*Seira domestica* Nicolet, 1842*	Yahyapour (2012), Yahyapour et al. (2011), Kahrarian et al. (2011), Daghighi (2012), Daghighi et al. (2013b)	Mazandaran/Sari, Kermanshah/Harsin, Gilan/Rasht	Leaf litter, Soil (*Magnolia* sp., *Ficus* sp., *Ulmus* sp., *Prunus* sp.)
**Subfamily Willowsiinae**			
**Genus *Willowsia* Shoebotham, 1917**			
*Willowsia nigromaculata* Lubbock, 1873*	Daghighi (2012), Daghighi et al. (2013b)	Gilan/Rasht	Soil (*Platanus* sp., *Prunus* sp., *Ulmus* sp., *Ficus* sp., *Alnus* sp., *Quercus* sp.)
**Subfamily Orchesellinae**			
**Genus *Heteromurus* Wankel, 1860**			
*Heteromurus major* (Moniez, 1889)	Cox (1982), Yahyapour (2012), Yahyapour et al. (2011), Daghighi (2012), Daghighi et al. (2013b)	Central, Mazandaran, Gilan, E. Azarbaijan, Mazandaran/Sari, Gilan/Rasht	Soil, Leaf litter
*Heteromurus nitidus* (Templeton, 1835)	Cox (1982)	Mazandaran, Gilan	Soil, Leaf litter
*Heteromurus sexoculatus* Brown, 1926	Cox (1982)	Mazandaran	Soil, Leaf litter
**Family Paronellidae**			
**Genus *Cyphoderus* Nicolet, 182**			
*Cyphoderus albinus* Nicolet, 1842*	Daghighi (2012)	Gilan/Rasht	Soil (*Platanus* sp.)
**Order Neelipleona**			
**Family Neelidae**			
**Genus *Neelus* Folsom, 1896**			
*Neelus murinus* Folsom, 1896	Cox (1982)	Central, Mazandaran, E. Azarbaijan	Soil, Leaf litter
**Order Symphypleona**			
**Family Sminthurididae**			
**Genus *Sphaeridia* Linnaniemi, 1912**			
*Sphaeridia pumilis* (Krausbauer, 1898)	Cox (1982), Kahrarian et al. (2012)	Central, Gilan Kermanshah/Sahneh/Harsin	Soil, Leaf litter
**Genus *Sminthurides* Börner, 1900**			
*Sminthurides malmgreni* (Tullberg, 1876)	Cox (1982)	Central, Gilan, E. Azarbaijan	Soil, Leaf litter
**Family Katiannidae**			
**Genus *Sminthurinus* Börner, 1901**			
*Sminthurinus aureus* Lubbock, 1862*	Yahyapour (2012), Daghighi (2012), Daghighi et al. (2013b)	Mazandaran/Sari, Gilan/Rasht	Leaf litter Soil (*Ulmus* sp., *Prunus* sp., *Acer* sp., *Cupressus* sp., *Robinia* sp., *Populus* sp.)
*Sminthurinus bimaculatus* (Axelson, 1902)	Cox (1982)	Gilan	Soil, Leaf litter
*Sminthurinus elegans* Fitch, 1863	Cox (1982), Yahyapour(2012), Falahati (2012), Ghahramaninezhad et al. (2012)	unknown, Mazandaran/Sari, Golestan/Gorgan, Kermanshah	Soil, Leaf litter, Leaf litter, Leaf litter
**Family Arrhopalitidae**			
**Genus *Arrhopalites* Börner, 1906**			
*Arrhopalites caecus* (Tullberg, 1871)	Cox (1982), Ghahramaninezhad et al. (2012)	Gilan, E. Azarbaijan, Kermanshah	Soil, Leaf litter
**Family Sminthuridae**			
**Genus *Sminthurus* Latreille, 1804**			
*Sminthurus viridis* Linnaeus, 1758*	Farrahbakhsh (1961)	Khuzestan	wheat and alfalfa fields
**Genus *Caprainea* Dallai 1970**			
*Caprainea marginata* (Schott, 1893)*	Kahrarian et al. (2012)	Kermanshah/Sahneh/Harsin	Soil
**Family Dicyrtomidae**			
**Genus *Dicyrtomina* Börner, 1903**			
*Dicyrtomina ornata* Nicolet, 1842*	Yahyapour (2012)	Mazandaran/Sari	Leaf litter
**Genus *Dicyrtoma* Bourlet, 1841**			
*Dicyrtoma minuta* (Fabricius, 1783)	Cox (1982)	Mazandaran, Gilan	Soil, Leaf litter
*Dicyrtoma fusca* Lubbock, 1873*	Yahyapour (2012)	Mazandaran/Sari	Leaf litter
